# Primary hepatoid carcinoma of the ovary: a case report

**DOI:** 10.11604/pamj.2015.20.93.5953

**Published:** 2015-02-02

**Authors:** Aicha Mazouz, Lamiae Amaadour, Hassania Ameurtesse, Laila Chban, Afaf Amarti, Fouad Kettani, Omar Addou, Siham Tizniti, Nawfel Mellas, Samia Arifi

**Affiliations:** 1 Department of Medical Oncology, Hassan II University Hospital, Fez, Morocco; 2 Department of Pathology, Hassan II University Hospital, Fez, Morocco; 3 Laboratory of Pathology, Avenue Nations Unies, Rabat, Morocco; 4 Department of Radiology, Hassan II University Hospital, Fez, Morocco

**Keywords:** Hepatoid carcinoma of the ovary, alpha-fetoprotein, immunohistochemical staining

## Abstract

Primary hepatoid carcinoma of the ovary (HCO) is a very rare type of high-grade invasive malignant ovarian tumor with hepatic differentiation and production of α-fetoprotein (AFP). We describe a 78-year-old Moroccan woman who presented to our hospital with abdominal distension and purplish nodules infiltrating the para umbilical skin with weight loss and impairment of her performance status. Excisional biopsy of the para umbilical nodule revealed a cutaneous localization of moderately differentiated adenocarcinoma and pelvic ultrasonography noted the presence of a tumoral right adnexal mass. The patient underwent an exploratory laparoscopy which found peritoneal carcinomatosis with pelvic adhesions allowing only a peritoneal biopsy. Diagnosis of primary hepatoid carcinoma of the ovary was established on the basis of classic histopathologic findings, immunohistochemical staining and marked elevation in serum of α-fetoprotein more than the carbohydrate antigen 125. The patient received 3 cycles of chemotherapy based on Carboplatin and Paclitaxel with disease progression. No second line chemotherapy was given because of the drop of patient's performance status to 3. The patient died one month later.

## Introduction

Primary hepatoid carcinoma of the ovary (HCO) is a very rare type of high-grade invasive malignant ovarian tumor with hepatic differentiation and production of α-fetoprotein (AFP) [1]. It is different from other ovarian neoplasms that have hepatic differentiation and production of AFP and must be distinguished especially from metastasis on the ovary of hepatocellular carcinoma (HCC) and hepatoid yolk sac tumors (HYST) [2]. The first case of primary HCO was described in 1987 by Ishikura and Scully [1]. To date, only 20 patients with diagnosis of primary HCO have been reported. We describe here the case of a 78-year-old Moroccan woman diagnosed with a primary HCO, the clinical and pathological characteristics are also briefly discussed.

## Patient and observation

**Figure 1:**
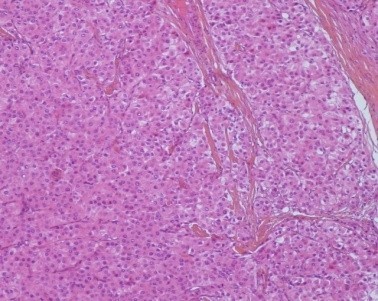
Histologic sections (HES× 100) of peritoneal biopsy showed polyhedral tumor cells with abundant eosinophilic cytoplasm, with a sinusoidal arrangement of vascular channels resembling liver without bile

**Figure 2:**
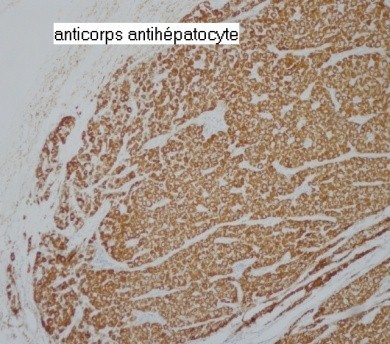
Immunohistochemical staining of the peritoneal biopsy showed a diffusely and overwhelmingly expression of the hepatocyte paraffin-1

A 78-year-old Moroccan woman with past medical history of Parkinson's disease presented to our hospital with an umbilical mass with loss of weight and impairment of the performance status (PS) in the last two months. The physical examination revealed an abdominal distension with purplish nodules infiltrating the para umbilical skin and the PS was scored 2. Nodule biopsy has been performed and revealed a cutaneous localization of moderately differentiated adenocarcinoma and pelvic ultrasonography noted the presence of a tumoral right adnexal mass. Her serum carbohydrate antigen 125 (CA125) level was 100 U/mL (normal <35 U/mL) and her serum AFP concentration was 150 ng/mL (normal <7.0 ng/mL). Her serum levels of carcinoembryonic antigen and CA-199 were within normal ranges. An exploratory laparoscopy was undertaken and found peritoneal carcinomatosis with pelvic adhesions allowing only peritoneal biopsies and there were no visible liver lesions with homogeneous appearance. Histologic sections of the biopsied peritoneal carcinomatosis showed polyhedral tumor cells with abundant eosinophilic cytoplasm, central round vesicular nuclei with variably prominent nucleoli in nests and trabeculae with a sinusoidal arrangement of vascular channels resembling liver without bile (Figure 1). The tumor was relatively homogeneous with no evidence of ovarian surface epithelial tumor morphology or germ-cell component. Paraffin tissue blocks were prepared and stained with antibodies to hepatocyte paraffin-1, AFP, cytokeratin 7(CK7), CK20, CK 8/18, S-100 protein, synaptophysin, inhibin, calretinin and epithelial membrane antigen. The results showed that tumor cells were positive for hepatocyte paraffin-1 which was diffusely and overwhelmingly expressed (Figure 2).

**Figure 3:**
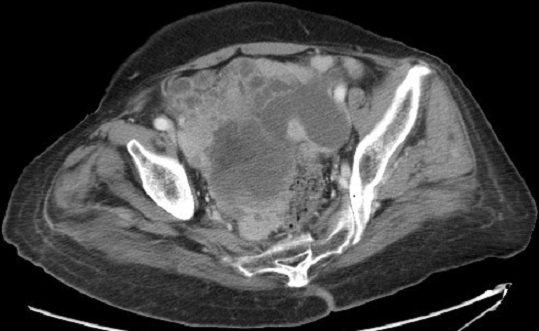
CT scan cut before chemotherapy showing peritoneal implants, right adnexal masse of 6.8 × 9.9 cm

**Figure 4:**
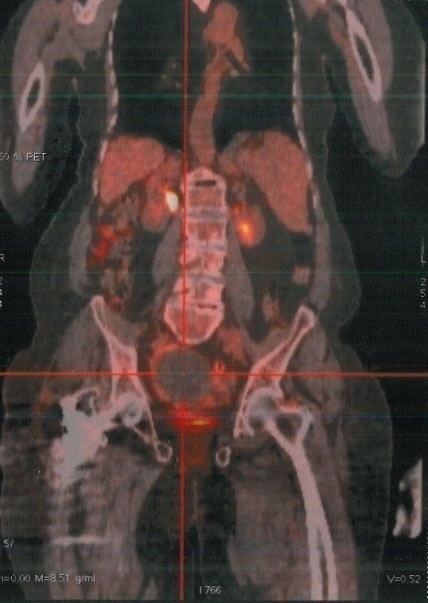
Positron emission tomography scans with FDG showing an intense hypermetabolisme in the pelvic (SUV max: 3.3) with no uptake in the liver

**Figure 5:**
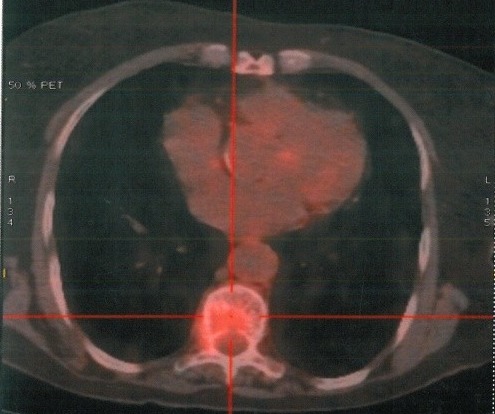
Positron emission tomography scans with FDG showing an intense hypermetabolisme in the eighth dorsal vertebra and pulmonary hilar nodes (SUV max: 3.8)

**Figure 6:**
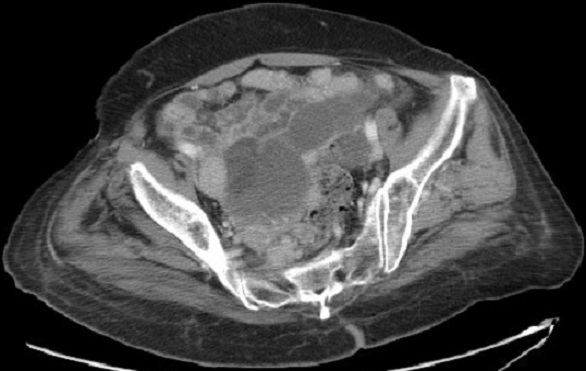
CT scan of the pelvis showing after 3 cycles of chemotherapy a stability according to RECIST 1.1

These hepatoid cells were positive for CK7 and CK8/18 but negative for CK20. Also, AFP, S-100 protein, synaptophysin, inhibin, calretinin and epithelial membrane antigen were all negative. A computed tomography (CT) scan of the pelvis showed an extensive omental peritoneal implants and bilateral adnexal masses with 6.8 × 9.9 cm on right (Figure 3) and 3.9 × 4.1cm on left. The patient got then after a positron emission tomography scan (PET/CT) with fludeoxyglucose (FDG) which revealed a metastatic disease with an intense hypermetabolisme in the pelvic with standard uptake value SUV max: 3.3 with no uptake in the liver (Figure 4). It revealed also an intense hypermetabolisme in the eighth dorsal vertebra with pulmonary hilar nodes (Figure 5). The Diagnosis of primary HCO was established on the basis of classic histopathologic findings, immunohistochemical staining and marked elevation in serum of α-fetoprotein more than the carbohydrate antigen 125. The disease was metastatic and the patient got a palliative chemotherapy with paclitaxel 175 mg/m2 and carboplatin with an area under the curve of 5 every 3 weeks. Evaluation after 3 cycles revealed the drop of patient's performance status to 3 versus 2 with an increase in the AFP level to 350 versus 150 ng/mL and radiologic stability according to RECIST 1.1(Figure 6). The patient was not legible for receiving a second line of treatment. She died one month later.

## Discussion

Primary HCO is a rare type of ovarian tumor which is similar to HCC histologically [1]. It is an aggressive tumor of uncertain origin which has been described in only post-menopausal women aged from 42 to 78 years with a mean age at diagnosis of 63 years [2]. The patient presents usually an abdominal distension and lower abdominal pain because HCO commonly presents at an advanced clinical stage and progresses rapidly with impairment of the general conditions and metastases to the abdomen and occasionally to the lungs; with median survival about 2 years [3]. Our patient was a post-menopausal woman with advanced clinical stage and PS scored at 2. Laboratory analysis revealed in HCO an elevation in serum CA 125 which is a marker for ovarian surface epithelial tumors. Also the serum AFP is elevated but more than the CA125, that suspicious a primary HCO. At diagnosis, our patient had marked elevation in serum of AFP more than the CA 125. A computed tomography scan of the abdomen showed in primary HCO a unilateral ovarian mass (rarely bilateral) which may appear on gross examination as entirely solid or with cystic areas and there may be multiple foci of hemorrhage and necrosis [2]. Our patient presented in CT scan bilateral ovarian masses but larger on the right. Microscopically, primary HCO resembles HCC with tumor cells that are arranged in sheets, cords and trabeculae. These cells have abundant eosinophilic cytoplasm and show frequent mitotic activity. Bile canalicular structures have rarely been described [4]. In our patient, histologic sections of the biopsied peritoneal carcinomatosis showed tumor cells with classic histopathologic findings in HCC without bile canalicular structures or ovarian surface epithelial tumor morphology.

The hepatoid differentiation of primary HCO is confirmed by expression in the immuno histochemical study of hepatocyte paraffin-1 which the degree of staining may also correlate with the degree of hepatoid differentiation. In our patient, hepatocyte paraffin-1 was diffusely and overwhelmingly expressed that suspicious a primary HCO without expression of AFP which can be sometimes focally expressed and consequently makes difficult to distinguish primary HCO from metastatic hepatocellular carcinoma in the ovary which is also a rare entity [5]. However they are biologically different, the CK profile of primary HCO resembles that of common epithelial adenocarcinoma which is CK19 and CK20 positive in primary HCO suggesting an epithelial origin for this entity and negative in CHC [2]. Also, the normal and neoplastic hepatocyte expresses diffusely the CK18 and doesn't express the CK7 which is expressing in primary HCO [6]. The CK profile of our patient was in favor of primary HCO with expression of CK7. A 2nd differential diagnosis of primary HCO is the HYST which is another ovarian tumor with hepatoid differentiation and AFP production but it is negative for hepatocyte paraffin-1. It occurs in younger women and we find a germ-cell tumor [2]. So it was unlikely that our patient had a HYST. Another differential diagnosis of HCO includes endometrial carcinoma, clear-cell carcinoma and lipid cell tumor which are all AFP negative [7]. To date, there is insufficient data regarding the optimal treatment of patients with primary HCO and most patients have been treated with ovarian-like cancer chemotherapy regimens, such as Carboplatine and paclitaxel, with short lived responses [8]. In second line, there is no therapeutic standard and given the pathologic resemblance of this tumor with HCC, Pandey M and Truica C reported a case treated by Sorafenib, by analogy to HCC, as second line chemotherapy with no response [9]. Our patient got a palliative ovarian chemotherapy as a first line of treatment with no response and no second line was permitted given her impairment of the PS at 3.

## Conclusion

Primary HCO is a rare highly aggressive tumor. Despite pathologic resemblance between primary HCO and HCC, they are biologically different and the primary HCO should be treated as an epithelial ovarian. However, most patients have been treated with ovarian-like cancer chemotherapy regimens with short-lived responses. The development of new therapies targeting this entity with poor prognosis is needed.
